# Earliest known funerary rites in Wallacea after the last glacial maximum

**DOI:** 10.1038/s41598-023-50294-y

**Published:** 2024-01-02

**Authors:** Stuart Hawkins, Gabriella Ayang Zetika, Rebecca Kinaston, Yulio Ray Firmando, Devi Mustika Sari, Yuni Suniarti, Mary Lucas, Patrick Roberts, Christian Reepmeyer, Tim Maloney, Shimona Kealy, Claudine Stirling, Malcolm Reid, David Barr, Torsten Kleffmann, Abhishek Kumar, Pratiwi Yuwono, Mirani Litster, Muhammad Husni, Marlon Ririmasse, Mahirta, Muhammad Mujabuddawat, Harriyadi, Sue O’Connor

**Affiliations:** 1 Archaeology and Natural History, School of Culture, History and Language, ANU College of Asia and the Pacific, Australian National University, Acton, ACT 2601 Australia; 2 ARC Centre of Excellence for Australian Biodiversity and Heritage, ANU College of Asia and the Pacific, Australian National University, Acton, ACT 2601 Australia; 3 Departemen Arkeologi Fakultas Ilmu Budaya, Universitas of Gadjah Mada, Yogyakarta, Indonesia; 4 Department of Anatomy, University of Otago, P.O. Box 913, Dunedin, 9054 New Zealand; 5 Griffith Centre for Social and Cultural Research, Griffith University, Nathan, QLD Australia; 6 BioArch South, Waitati, 9085 New Zealand; 7 Department of Archaeology, Max Planck Institute of Geoanthropology DE, Jena, Germany; 8 isoTROPIC Research Group, Max Planck Institute of Geoanthropology, Jena, Germany; 9 Commission for Archaeology of Non-European Cultures, German Archaeological Institute Division of Germany, Berlin, Germany; 10 ARC Centre of Excellence for Australian Biodiversity and Heritage, College of Arts, Society, and Education, James Cook University, Cairns, QLD 4870 Australia; 11 Griffith Centre for Social and Cultural Research, Griffith University, Southport, QLD 4222 Australia; 12 Centre for Trace Element Analysis, Department of Geology, University of Otago, Dunedin, 9054 New Zealand; 13 Centre for Protein Research, Department of Biochemistry, University of Otago, Dunedin, 9054 New Zealand; 14 Geoarchaeology and Archaeometry Research Group (GARG), Southern Cross University, Lismore, NSW Australia; 15 Balai Arkeologi Maluku, JI. Namalatu-Latuhalat, Ambon, Indonesia; 16 Organisasi Riset Arkeologi Bahasa dan Sastra, Badan Riset dan Inovasi Nasional, Jakarta, Indonesia

**Keywords:** Palaeoecology, Stable isotope analysis, Archaeology, Cultural evolution, Social evolution, Evolution, Ecology, Environmental sciences, Ecology, Climate-change ecology, Palaeoecology, Stable isotope analysis

## Abstract

The insular region of Wallacea has become a focal point for studying Pleistocene human ecological and cultural adaptations in island environments, however, little is understood about early burial traditions during the Pleistocene. Here we investigate maritime interactions and burial practices at Ratu Mali 2, an elevated coastal cave site on the small island of Kisar in the Lesser Sunda Islands of eastern Indonesia dated to 15,500–3700 cal. BP. This multidisciplinary study demonstrates extreme marine dietary adaptations, engagement with an extensive exchange network across open seas, and early mortuary practices. A flexed male and a female, interred in a single grave with abundant shellfish and obsidian at Ratu Mali 2 by 14.7 ka are the oldest known human burials in Wallacea with established funerary rites. These findings highlight the impressive flexibility of our species in marginal environments and provide insight into the earliest known ritualised treatment of the dead in Wallacea.

## Introduction

The arrival of anatomically modern humans (AMH) into the insular region of Wallacea ca. 46–40 ka^1–4^ constitutes some of the earliest maritime adaptations of our species^5–10^. These adaptations occurred during substantial climate-mediated sea level fluctuations that influenced island size and ecologies, as well as human migration and adaptation to island environments^5,11–16^. Wallacea lies between the continental shelves of Sunda and Sahul, to which it has never been connected, and contains the islands of eastern Indonesia (Sulawesi, Lesser Sunda Islands, and the Moluccas) and the independent country of Timor-Leste on Timor^12,13^. Wallace’s line refers to the biogeographic break separating Wallacea from Sunda to the West and is situated between Borneo and Sulawesi and between Bali and Lombok across which the dispersal of Asian biota was restricted^12,13^. These unique biogeographical conditions, with some of the highest marine biodiversity on the planet^17^ and restricted terrestrial biodiversity relative to Sunda and Sahul, likely contributed to the emergence of biological, cultural, and economic variability seen in contemporary societies in the region today^18–21^. Studying mortuary practices has become an important research avenue to understand past variability in human diet and ritualised behavior in Island Southeast Asia (ISEA) and the Pacific^22–25^. However, the emergence of early Wallacean mortuary practices within this insular biogeographic context is largely unknown due to a scarcity of Pleistocene burial discoveries^26,27^.

**Figure 1:**
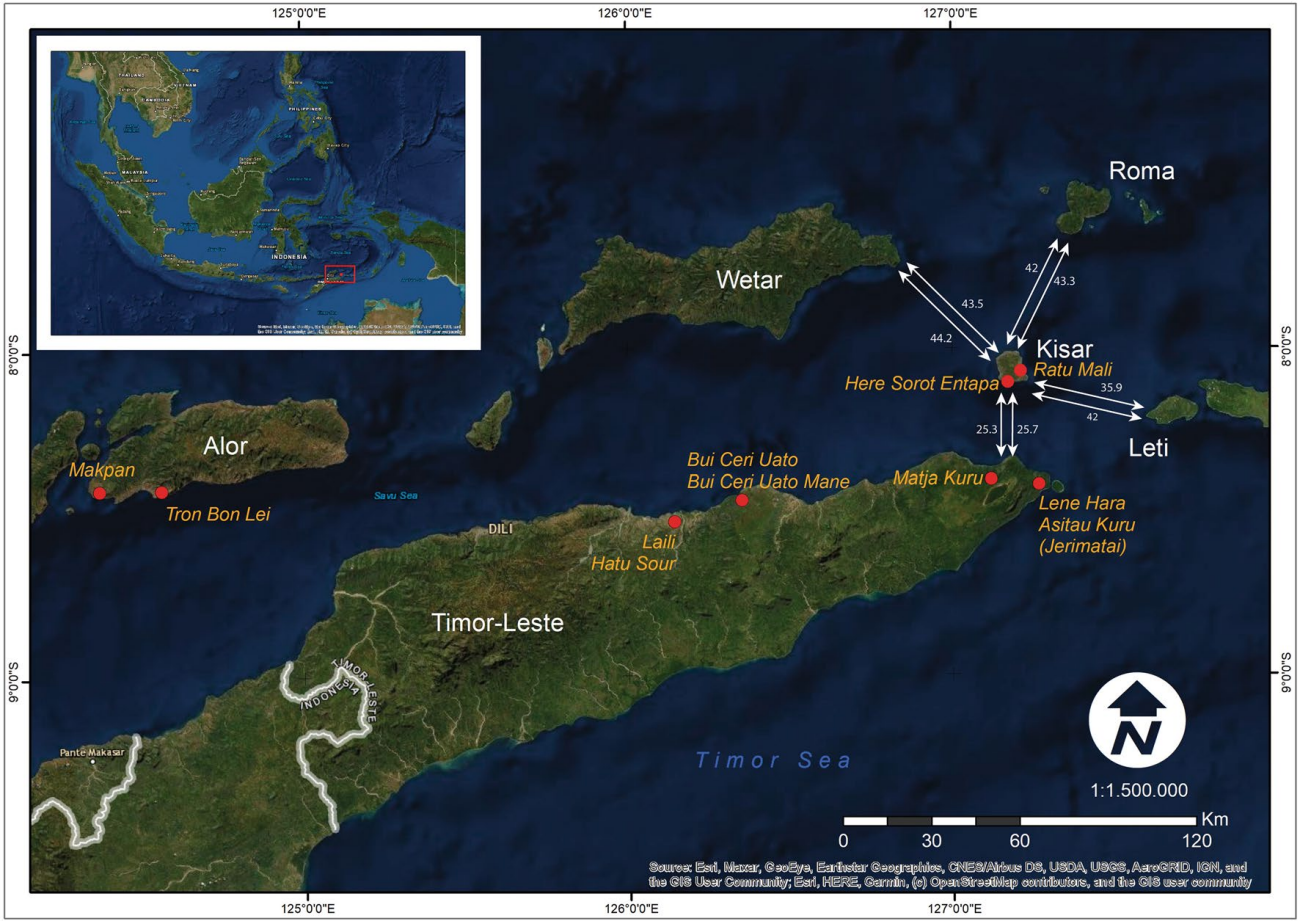
Location of Ratu Mali 2 (RM2), and Here Sorot Entapa (HSE), Kisar Island, eastern Indonesia. ArcGIS Desktop version 10.7.0.10348. https://desktop.arcgis.com/en/quick-start-guides/10.7/arcgis-engine-developer-kit-and-engine-quick-start-guide.htm.

**Figure 2:**
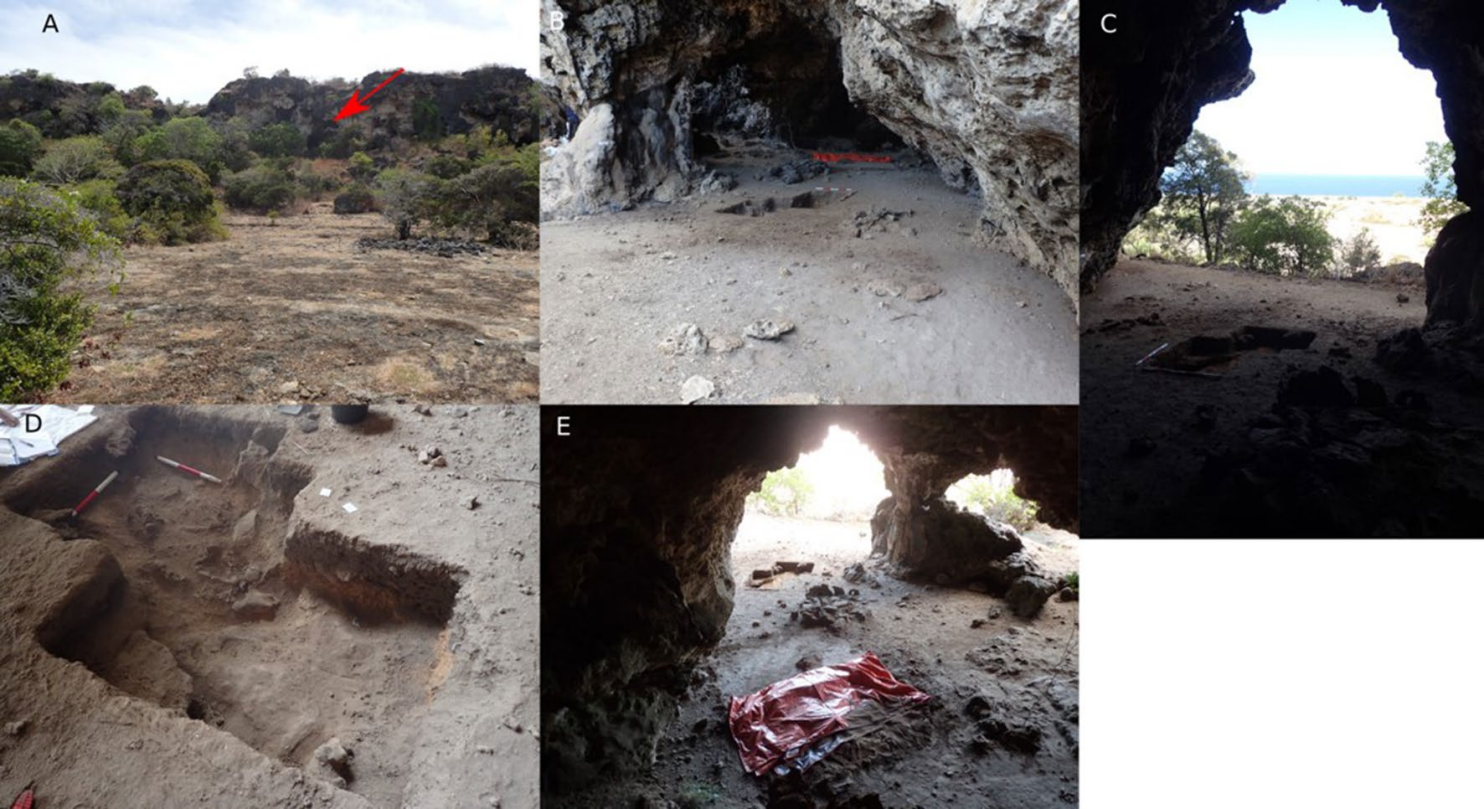
Ratu Mali 2; (**A**) View of Ratu Mali 2 from the coastal flat facing west; (**B**) Ratu Mali 2 and excavation area facing west; (**C**) Ratu Mali 2 cave opening facing east towards the coast; (**D**) Excavation units SQ A, SQ B ext, and unit C ext; (**E**) Ratu Mali 2 facing east towards the cave opening.

Here, we investigate AMH interactions with marine environments in the Lesser Sunda Islands of Wallacea over several millennia^1–3,5–10^ and the emergence of mortuary practices after the last glacial maximum (LGM) ca. 25–18 ka^28^, when rising sea levels, and generally warmer and wetter conditions^29–31^, coincided with expanding populations^32^, the development of large-scale maritime exchange networks and new technology^33–37^. We focus on our recent significant findings at the coastal cave site, Ratu Mali 2 (RM2) (Figs. 1, 2), on the small island of Kisar, eastern Indonesia, which includes evidence of the earliest mortuary practices so far discovered in the insular region of Wallacea 15.5–14.7 ka. We reconstruct maritime interactions utilizing archaeological evidence (chronostratigraphic modelling of radiocarbon dates, faunal analyses, artefact analyses, obsidian geochemical characterisation) from a sequence spanning 15.5–3.7 ka. Post-LGM burial practice, chromosomal sex, diet, and childhood residency of two individuals interred in a single grave feature was explored through field anthropology, osteological analysis, grave goods, and isotopic and enamel peptide analyses.

### Ratu Mali 2 site and chronology

Kisar is a small island, with an area of 81.15 km^2^ (Fig. 1). Due to its steep offshore bathymetry, land area would have remained relatively consistent in size since human settlement commenced ~ 15.5 ka^38^. Kisar is part of the active Banda arc-continent collision zone and has a metamorphic core ringed by both emerged and submerged limestone terraces^39^. The island is visible from Timor-Leste 25 km to the southwest and has a narrow reef platform that is overlooked by uplifted coralline limestone cliffs with few coastal access points. Kisar is drier and relatively more impoverished in terrestrial fauna (small rat, bat, snake, lizard, and bird species) compared to the larger islands in the region^38,40^. The current vegetation is dry savannah with woodland patches and the island has few seasonal freshwater sources and low rainfall, which falls mostly during the monsoon season from November to May^38^. Settlement on Kisar would thus have been vulnerable to the climatic shifts affecting water and faunal resources that occurred in Wallacea since the LGM^29–31^.

**Figure 3:**
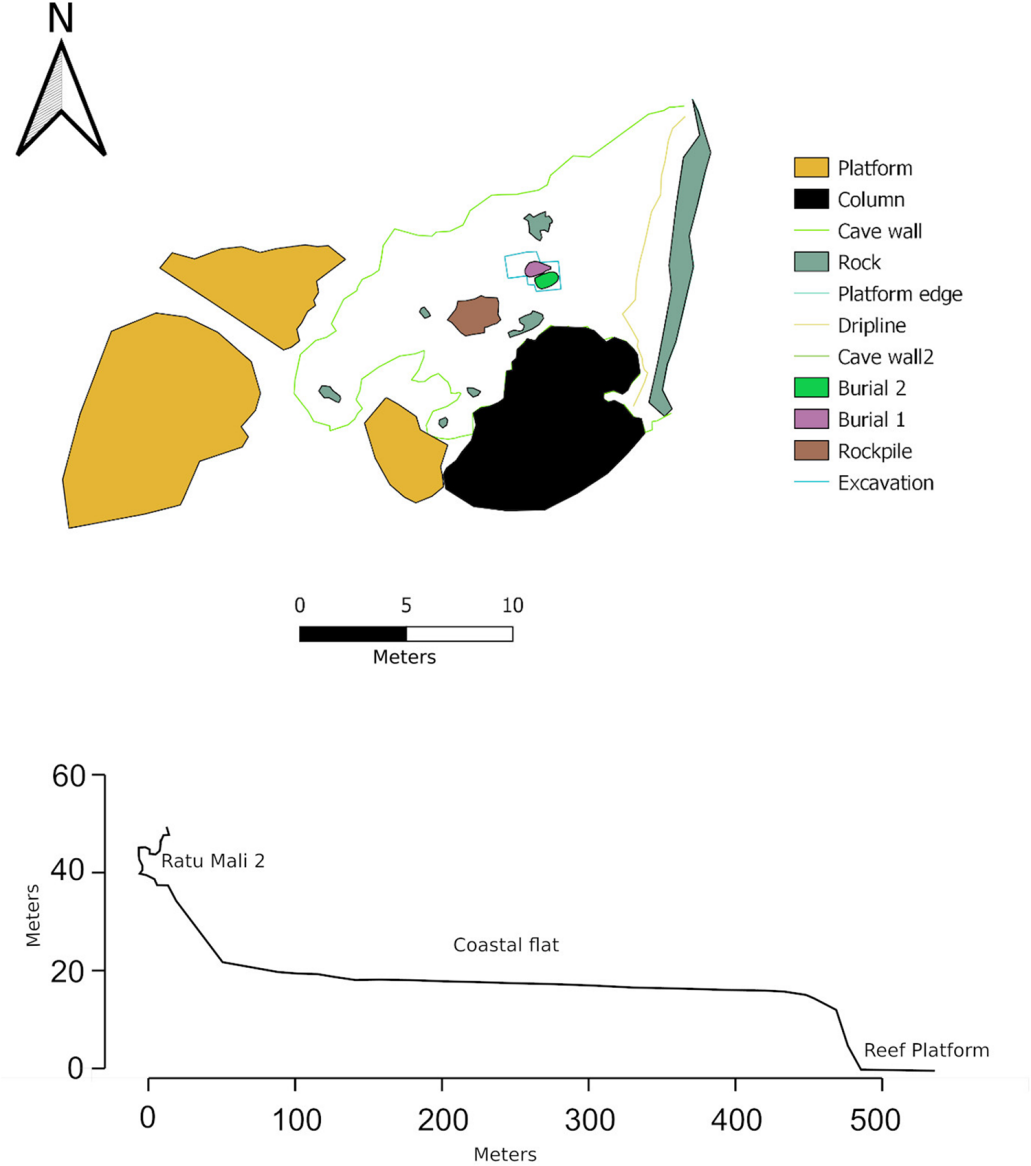
Ratu Mali 2, excavation area, and site plan (above) and transect from cave to sea level (below).

**Figure 4:**
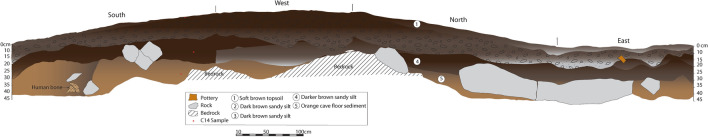
SQ A and SQ B extension Section drawing.

**Figure 5:**
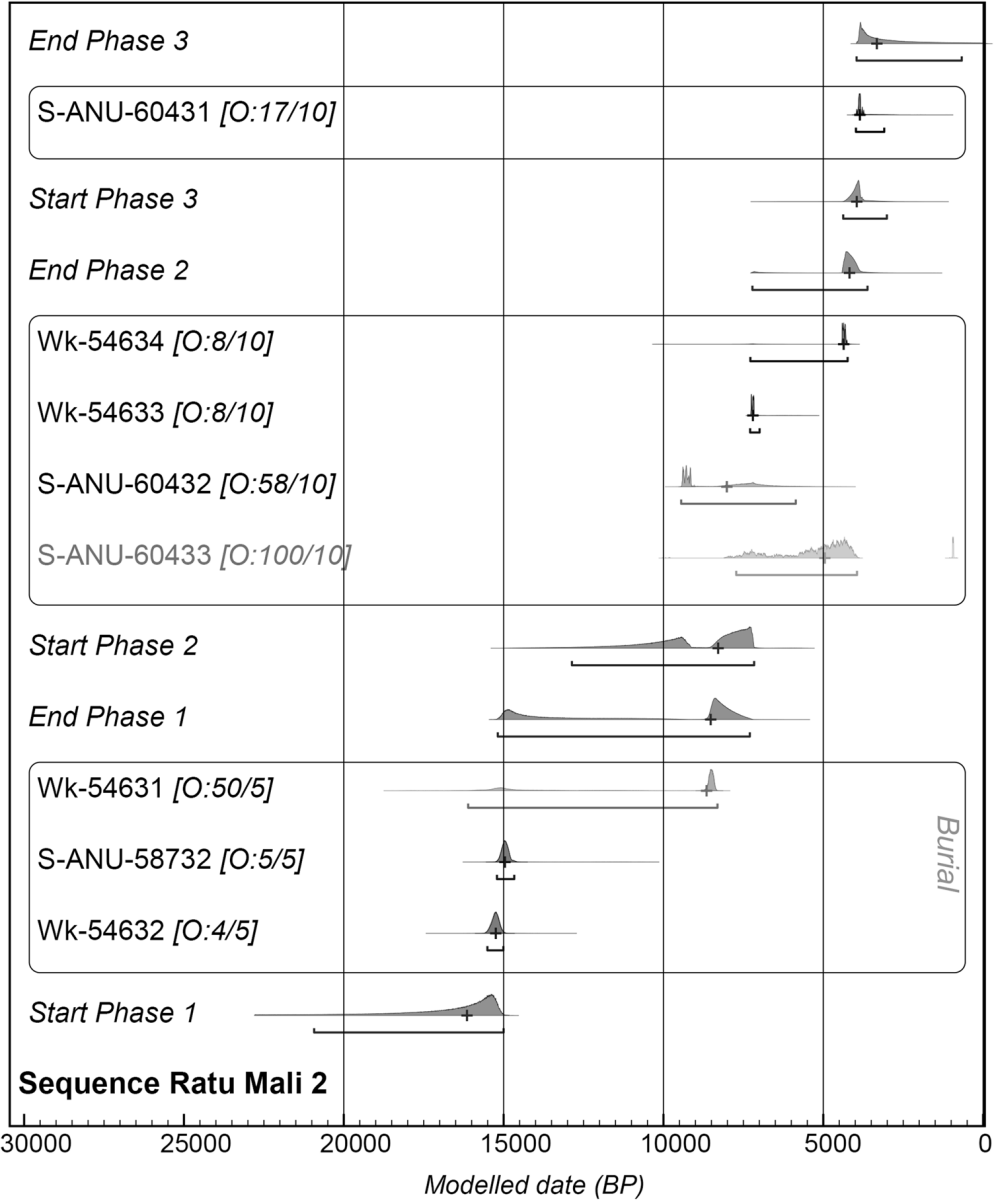
Bayesian chronostratigraphic model for Ratu Mali 2.

**Figure 6:**
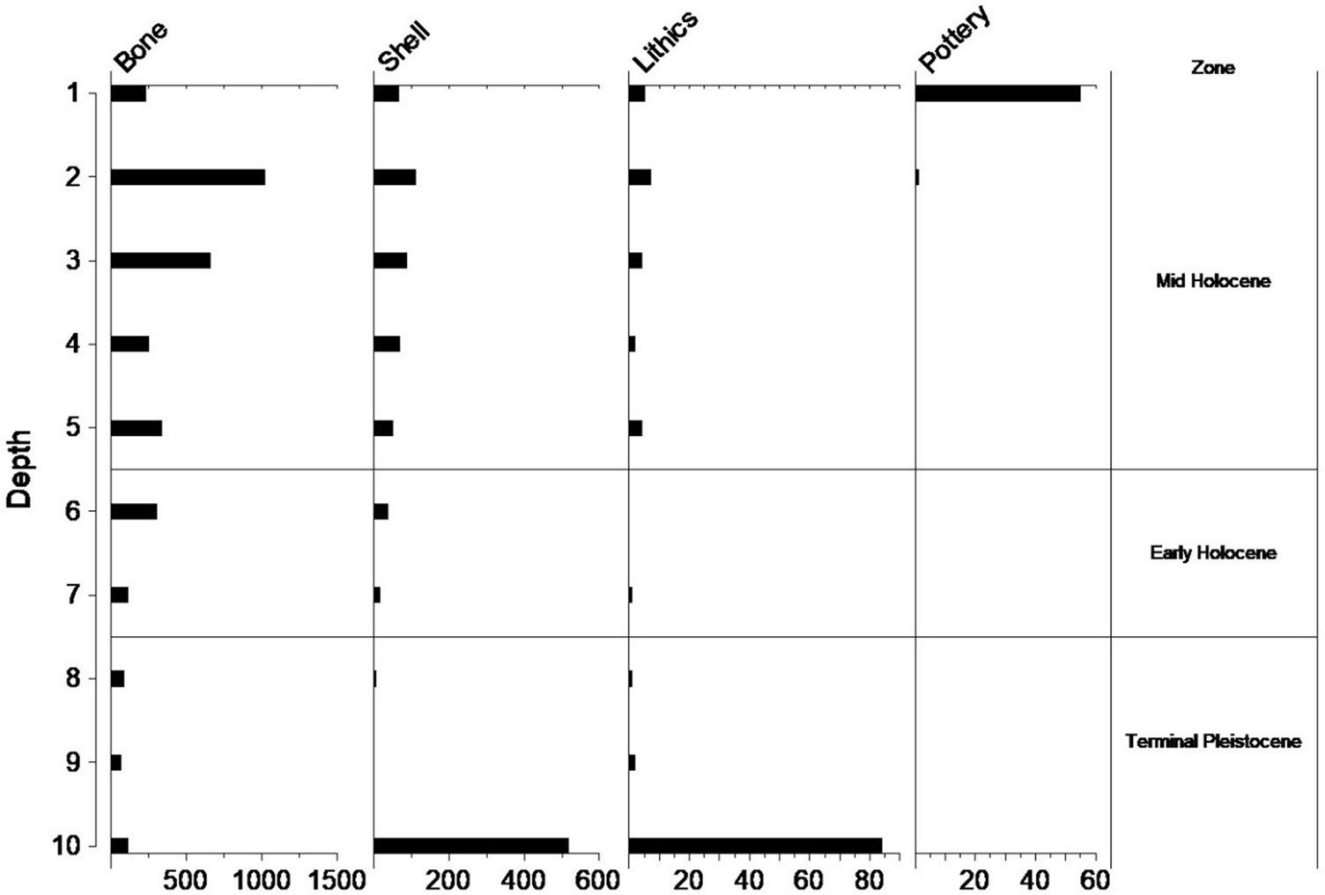
Ratu Mali 2 materials by spit and burial. Depth 1–9 = A1–A9, 10 = burial feature. NISP for bone, lithics, pottery, MNI for shell in x-axis.

RM2 is located 500 m inland from the east coast of Kisar in the upper limestone coastal terrace elevated at 38m asl, and 650 m to the south of Jawalan harbor, which provides access for watercraft and reef foraging (Figs. 1, 2, 3). RM2 is a modest-sized cave 40 × 20 m, with a gently sloping floor, high ceiling (ca. 10 m), and large opening overlooking the surrounding coastal terrace below (Figs. 2, 3). A constructed rock cairn adjacent to the excavation (Figs. 2, 3), indicates that at some time the site held socio-cultural significance. SQ A (1 m^2^) was excavated in nine 5 cm spits (A1–9) near the cave entrance to a depth of 45–55 cm until bedrock was encountered. An additional 100 × 50 cm extension (SQ B) at the eastern side of SQ A was excavated to expose a basal grave feature, and six 5 cm spits (B1–6) were excavated down to the top of the grave feature. Due to time constraints, the pit was also extended without squares to the southwest to expose the rest of the grave feature. Five layers (Fig. 4) were recorded in the field based on colour and sediment consistency and the chronostratigraphic model which incorporates the eight radiocarbon dates (Fig. 5; Supplementary Tables 1–3) suggests three major phases from which cultural materials were recovered (Fig. 6, Supplementary Tables S1–17).

Phase 1 represents the initial use of the cave to inter the dead where two poorly preserved individuals were recorded in situ in a single grave. The chronostratigraphic model provides a median estimate of ~ 16 ka for the start of Phase 1 and an end date between ~ 15–7 ka (95% probability) (Fig. 5, Supplementary Tables S2–3) depending on whether or not the inverted date Wk-54631 is included. The grave appears to have been dug into Layer 5, a natural orange brown layer with sparse cultural material, to a maximum depth of 45–50 cm to the bedrock shelter floor. The grave cut could be clearly seen in section and in plan and could be excavated as a discrete feature. Direct dating of an abalone (*Haliotis varia*) and a chiton (*Acanthopleura* spp.) sample adjacent to Burial 2 in the grave fill base returned dates of 15,179–14,725 cal. BP (S-ANU-58732, 13,129 ± 31 BP) and 15,504–15,043 cal. BP (Wk-54632, 13,373 ± 39 BP) respectively, indicating that this burial was contemporaneous with the earliest phase of Kisar settlement at Here Sorot Entapa (HSE)^38^, 5.8 km southeast of RM2 on the south coast.

**Figure 7:**
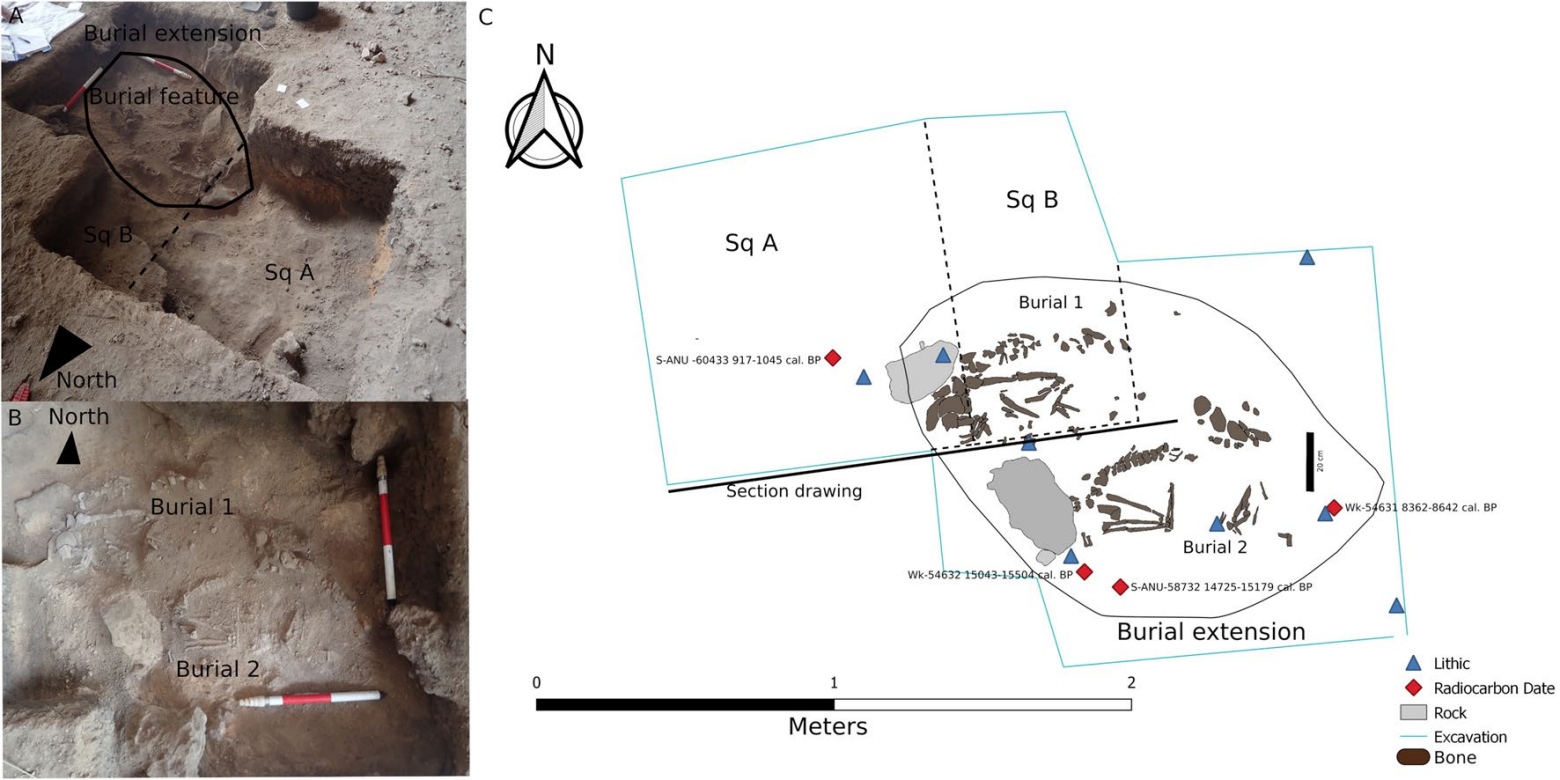
(**A**); Excavation unit including SQ A, SQ B, burial extension and burial feature: (**B**); Burials 1 and 2 photograph: (**C**); Digital excavation plan including grave feature at Ratu Mali 2 with 3D recorded radiocarbon and lithic samples associated with burial 1 and 2.

Two inverted dates were also recorded in the basal deposits raising the possibility that the samples acquired are not from discrete chronostratigraphic phases. A single inverted charcoal radiocarbon date (S-ANU-60433, 1042 ± 23) of 964–914 cal. BP was obtained in SQA Spit 9 near the base of Layer 5 where intrusive burnt tree roots were also recorded. The chronostratigraphic model indicates that this inverted date has a 100% probability of being an outlier and is thus justifiably removed from further chronostratigraphic considerations (Fig. 5, Supplementary Tables S2–3). An abalone shell (*Haliotis varia*) (Wk-54631, 8199 ± 25) dating to 8642–8362 cal. BP was retrieved near the edge of the burial cut (Fig. 7) and higher up in the fill than the two dates recovered from the base of the burial. While identified as associated with the burial during recovery, and modelled within this initial burial phase for posterity, the proximity of this sample to the contact point between two discrete provenance units suggest it might belong to Layer 5 or 4.

Supporting this scenario is the similar date of 9419–9135 cal. BP (S-ANU-60432, 8291 ± 31) obtained from a charcoal sample in Layer 5, near the Layer 4/5 interface. Given the seemingly sterile nature of Layer 5, it is most likely that this date, as well as the few cultural materials associated with Layer 5, are in fact the result of post-depositional vertical movement from Layer 4, and/or slight mixing of layers along their contact boundary during excavation. To account for this, Layer 5 was modelled alongside Layer 4, within the second phase of occupation (Fig. 5, Supplementary Tables S2–3).

The chronostratigraphic model places the beginning of Phase 2 at 8 ka and ending at 4 ka dating this phase to the early to mid-Holocene. This phase is characterized by sparse chert artefacts, animal bone and mollusc remains in Layers 4 and 3. Two charcoal radiocarbon dates of 7268–7165 cal. BP (Wk-54633, 6306 ± 15) and 4419–4291 cal. BP (Wk-54634, 3919 ± 16) were obtained from Layer 4 in Phase 2.

Phase 3 covers the Late Holocene period starting at 4 ka and ending at 3 ka (Fig. 5, Supplementary Tables S2–3). It contains more concentrated lithic, animal bone and shell material relative to Phase 2. A single charcoal radiocarbon date 3967–3729 cal. BP (S-ANU-60431) in Layer 2 (Spit 2) places the Phase 3 deposits well within the early Neolithic period of ISEA postdating 4200 cal BP, and characterised by pot-making peoples with domesticated animals^41–43^. The Phase 3 deposits contained concentrated white/grey ash lenses and abundant charcoal and are clearly distinct from the early Holocene deposits of Phase 2. Phase 3 also contained pottery fragments, found mostly in the top Spit 1. The site was abandoned soon after but may have been used in the recent past for goat herding activities.

The chronostratigraphic model does not reveal an occupation hiatus in the RM2 sequence if Wk-54631 is included in Phase 1. However, since we have argued that there is a high possibility that it does indeed belong in Phase 2, then our dates would suggest a slight chronological break between Phases 1 and 2 (Fig. 5, Supplementary Tables S2–3).

### Mortuary practices and maritime interactions at Ratu Mali 2

**Figure 8:**
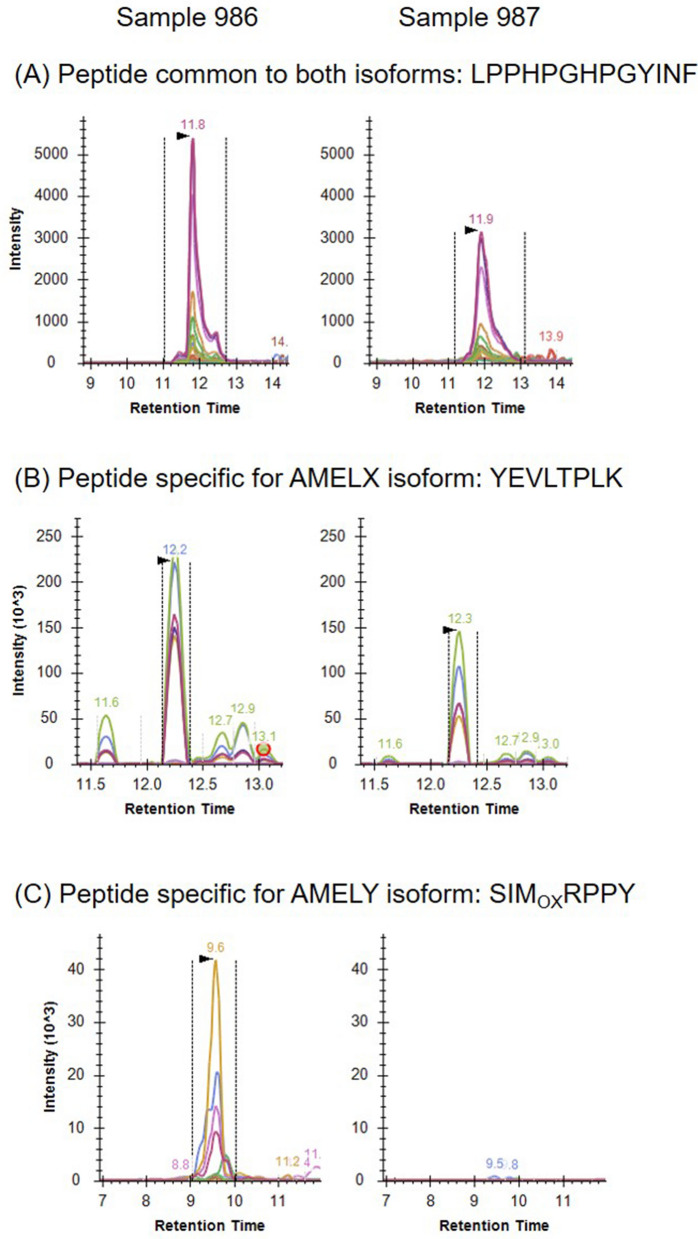
Fragment ion intensities measured by high resolution multiple reaction monitoring. For both samples (986 and 987) a set of peptide precursor ions was selected that are either present in (**A**) both the female and male isoform AMELX and AMELY, respectively, or are specific for (**B**) the female isoform or (**C**) the male isoform. Ion signals for the male isoform AMELY were detected in high intensities in sample 986 (Burial 1) but not in sample 987 (Burial 2) identifying burial 1 as male and Burial 2 as female.

The two RM2 individuals were interred in a single shallow grave, 90 cm in diameter, in a primary burial position directly on top of the natural bedrock 45–55 cm below the ground surface. The burials were oriented along an east–west axis facing south towards Timor-Leste and large limestone rocks were placed near the head of each burial (Fig. 7). Both RM2 individuals were found in identical flexed position on their right sides, with hands carefully tucked under the head (Burial 1) or chin (Burial 2) (Fig. 7). Less than 50% complete, the skeleton of Burial 1 had a highly fragmented cranium but most teeth were intact, as were the ribs and hands, while the lower body was less than 10% complete (Supplementary Table S4). Burial 2’s cranium was more fragmentary than Burial 1 with only 20% of teeth present, but had 60% of hands, and 30% of legs intact (Supplementary Table S4). Enamel peptide analysis revealed Burial 1 was chromosomally male and Burial 2 was chromosomally female (Fig. 8, Supplementary Table S5) and represent the oldest known individuals to be sexed using this method.

**Figure 9:**
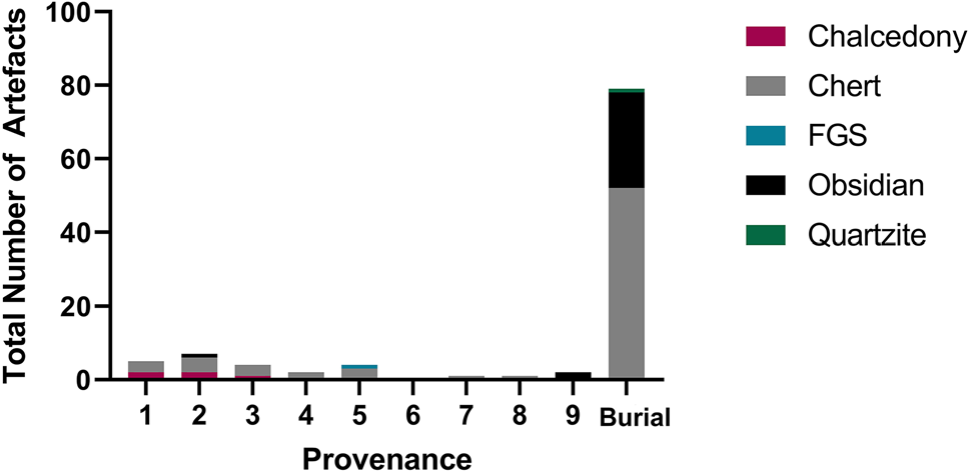
Total number of lithic artefacts by raw material and spit for square A (A1–A9).

**Figure 10:**
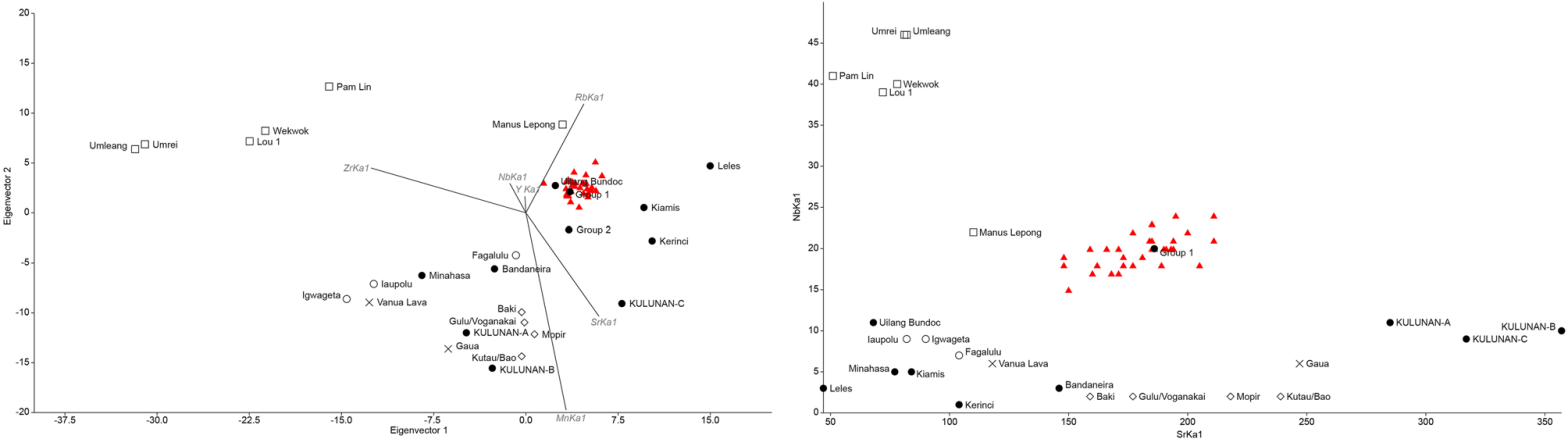
LDA and Bplot for obsidian sources at Ratu Mali 2 compared to other sites in Island Southeast Asia.

The grave fill of the interred in Phase 1 is associated with most of the obsidian flakes (3.7–21.2 mm in percussion length) recovered from RM2 (Phase 1 n = 26, Phase 2 n = 4, Phase 3 n = 1) (Fig. 9; SI 6–7). The obsidian in the RM2 grave fill, as well as in Phase’s 2 and 3, derives from a single exotic geochemical source which has been designated Group 1^38^ (Fig. 10). Strontium isotope data (^87^*Sr*/^86^*Sr*) from the buried individuals at RM2 may indicate they spent their childhood on Kisar or another island with a similar limestone geology. Both individuals displayed tooth enamel ^87^Sr/^86^Sr values (Burial 1, 0.70985; Burial 2, 0.70951) (Supplementary Table S8) that were slightly elevated compared to the worldwide ^87^Sr/^86^Sr value of seawater (0.7092) and, therefore, limestone. The human ^87^Sr/^86^Sr values were similar to the modern goat tooth enamel ^87^Sr/^86^Sr values (0.70979 and 0.70924) (Supplementary Table S8), supporting that both spent their childhood on Kisar or an island with a similar underlying geology. It should be noted that ‘marine’ strontium isotope signatures in human tooth enamel may be influenced by one or more of the following factors^44^: (1) the influence of the sea spray effect^45^ that causes the deposition of ocean-derived ^87^Sr/^86^Sr values obscuring inputs from the terrestrial environment; (2) consumption of resources grown or living on the underlying limestone, which displays similar values to marine strontium^46^; (3) the consumption of marine foods^47^.

**Figure 11:**
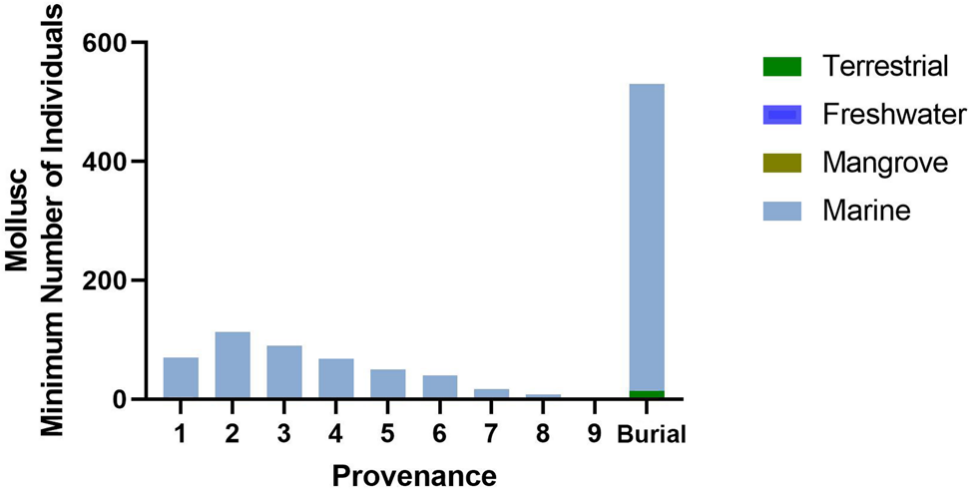
Ratu Mali 2 shell taxa by spit and habitat for Square A.

**Figure 12:**
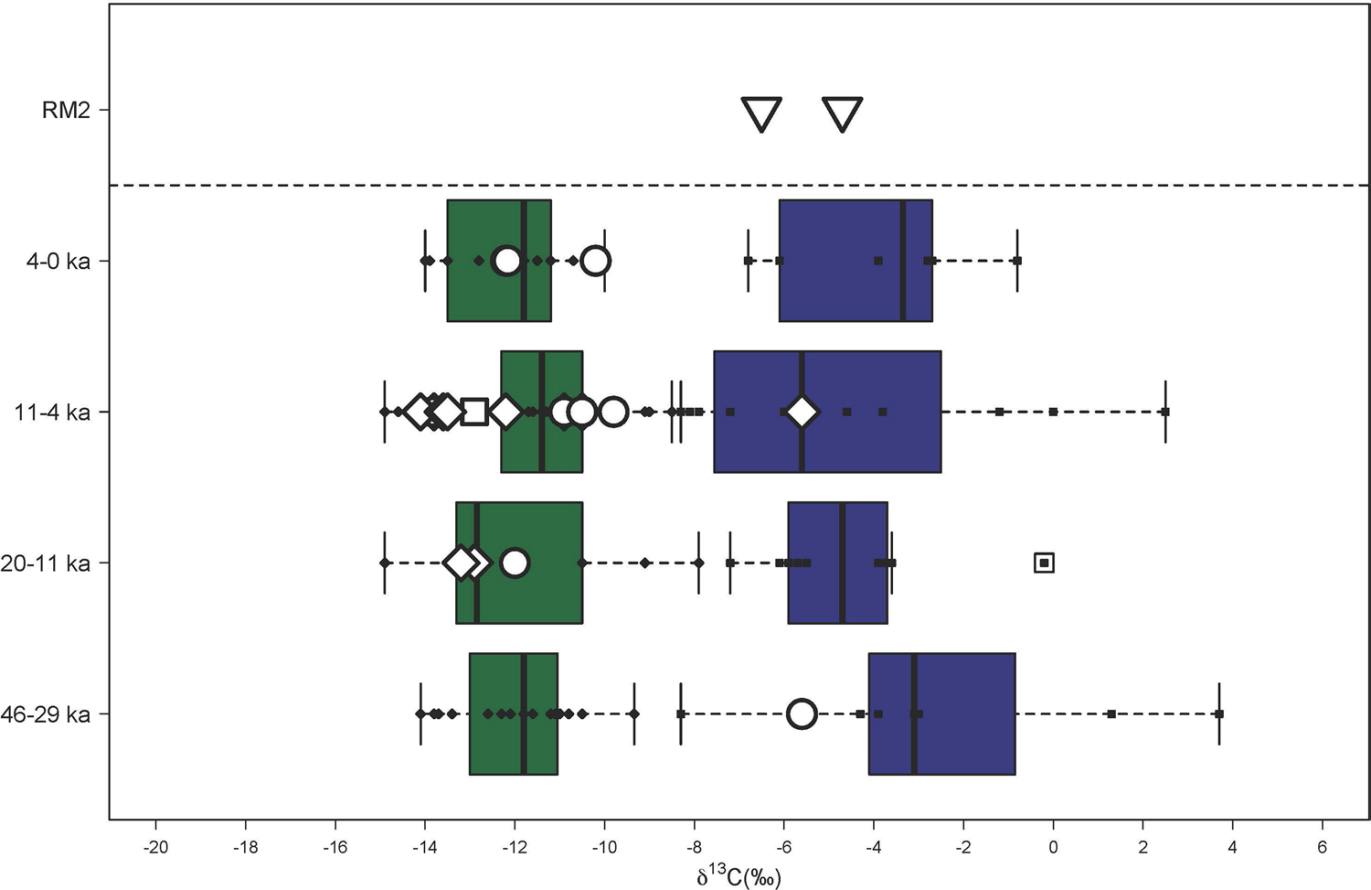
Carbon and oxygen isotope data from human teeth samples associated with the two Pleistocene individuals buried at Ratu Mali 2 (RM2) in relation to other human teeth from sites in Wallacea after Roberts et al*.* 2020.

**Figure 13:**
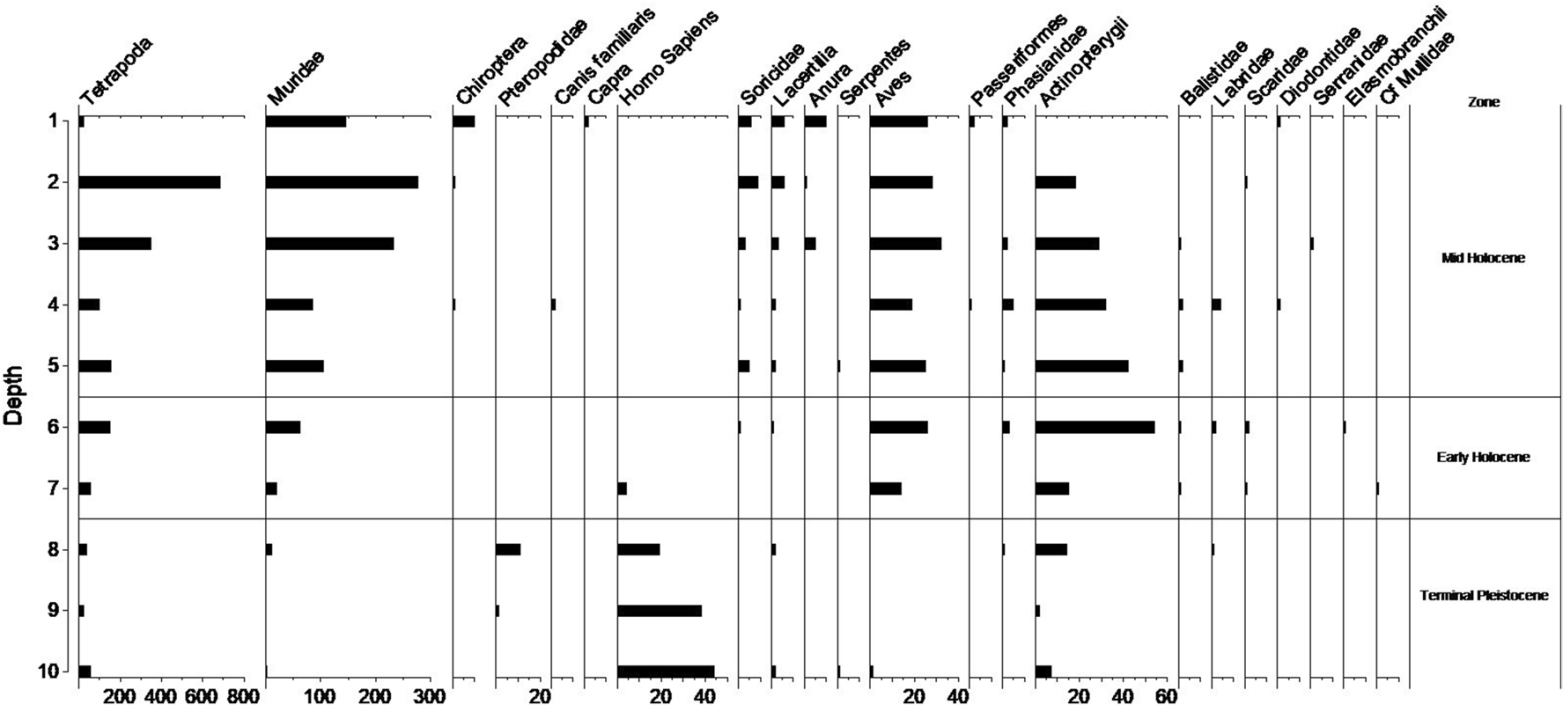
Ratu Mali 2 vertebrates for Square A by spit (A1–9) and burial^10^.

The importance of rocky reef shellfish foraging since the Terminal Pleistocene at RM2 is evident based on the recovery of large quantities of molluscs from this habitat throughout the sequence (Fig. 11, Supplementary Tables S9–10). Heavy reliance on marine resources is also supported by stable carbon isotopic analyses of the teeth of the two individuals interred at RM2 (Fig. 12, Supplementary Table S8), although there is a small chance this may also indicate wild C_4_ resources^23^. Based on relative abundance, marine shellfish was significantly more concentrated in the burial fill of Phase 1 relative to subsequent Phases 2 (early Holocene) and 3 (Late Holocene) (Phase 1; MNI = 530, Phase 2; MNI = 161, Phase 3; MNI = 275). The high diversity of marine shellfish (Number of Taxa = 89) at RM2 indicates a generalist reef gleaning strategy as well as a healthy and vibrant littoral reef zone. The evenness diversity index (Equitability J), which takes into account taxonomic relative abundance, indicates that foraging was focused more evenly on a wide range of shellfish resources in later Phases 2 and 3 but appeared more specialised and focused on fewer taxa in the burial fill of Phase 1 (Phase 1 = 0.5067, Phase 2 = 0.6081–0.7972, Phase 3 = 0.6859–0.8447) (Supplementary Table S11). Foraging was focused on rocky shore families Neritidae and Chitonidae making up 83% of the total MNI recovered. Shellfish abundance also increased over time from Phase 2 to Phase 3. However, there was no notable change in diversity, evenness, and equitability during this period, suggesting significant increases in foraging intensity by the Late Holocene but no significant changes in foraging behavior. While shellfish gleaning appears to be the most dominant subsistence behaviour practiced at RM2 throughout the sequence, small amounts of marine finfish (Actinopterygii) were present (Fig. 13, Supplementary Tables S12–13). Crab (Decapoda) carapace fragments were also found in small numbers (Supplementary Table S14). Most crab remains occur in the upper levels, and may represent post-depositional disturbances closer to the surface.

Prehistoric terrestrial resource exploitation on Kisar is difficult to assess. Most of the vertebrate remains deposited at RM2 were tetrapod microfauna (small rodents, lizards, frogs, snakes, birds, and bats) (Fig. 13, Supplementary Tables S12–13). Based on other studies conducted in Wallacea, they likely represent remains deposited by avian predators^48,49^. Megafauna and giant rats, found on other islands in Wallacea^1–4,49,50^, were not present on the small island of Kisar at the time of human settlement. At RM2, changing terrestrial interactions are likely related to Neolithic dispersal as indicated by the small numbers of introduced dog and goat bones (Fig. 13, Supplementary Tables S12–13) in association with pottery (Supplementary Table S15) which we recovered from the upper Late Holocene deposits of Phase 3.

**Figure 14:**
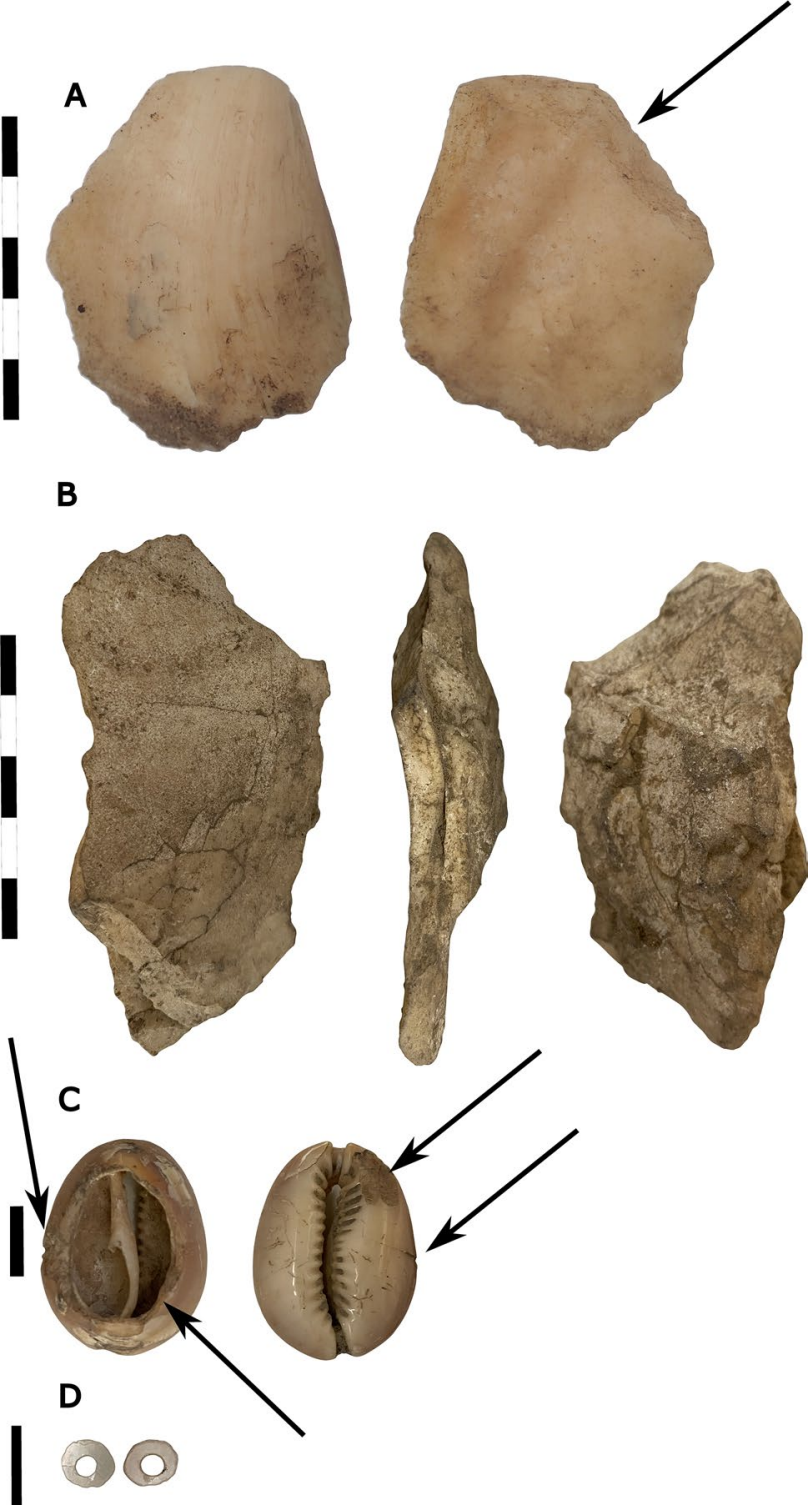
Shell artefacts from Ratu Mali 2, arrows indicate percussion impact scars and zones of modification; (**A**) Ground Tridacna flake, A5 Layer 4 mid-Holocene,5 cm scale bar; (**B**) *Tridacna* flake, A2 Layer 2 Neolithic, 5 cm scale bar; (**C**) Modified cowrie (Cypraeidae) shell with dorsum removed, associated with Pleistocene burial feature, 1 cm scale bar; (**D**) *Nautilus* shell disc-bead, single hole variety, A2 Layer 2 Neolithic, 1 cm scale bar.

Much like the HSE lithic assemblage^38^, we recovered few formal tools at RM2, and mostly un-retouched flakes that share similarities with other lithic assemblages in Wallacea^1,51^. RM2 lithic artefacts (N = 126, Fig. 10, Supplementary Tables S9–10), varying between 2.4 and 43.7 mm in percussion length, comprised mostly chert and obsidian flakes, with smaller amounts of quartz, quartzite, a fine-grained silicified limestone and chalcedony. Other cultural materials were recovered from RM2. Shell disc-bead ornaments made out of *Nautilus sp*. of the single-hole variety (Fig. 14, Supplementary Table S16), were recovered in the Late Holocene deposits of Phase 3. These are similar to those found on Timor^52–54^, Alor^2^, and at HSE on Kisar^38^. Two *Tridacna* adze blanks were recovered, one from Phase 2 and one from Phase 3, revealing evidence for adze manufacture at RM2 since the early Holocene (Fig. 14, Supplementary Table S16).

## Discussion and conclusions

Mortuary practices in ISEA are not well known for the Pleistocene^26,27^, although AMH remains are present in the earliest archaeological sites dating back to 73 ka in Sumatra^55^. The earliest burial in ISEA is a flexed primary burial with evidence for amputation dated to 31.5–30 ka at Liang Tebo on Borneo^56^, to the west on the Sunda shelf. Also on the Sunda shelf, the Gua Braholo 6 burial on Java, dated to 15,371–12,698 cal. BP (P3G-1998, 13,290 ± 400), overlaps in age with the RM2 burials^57^. The two primary flexed individuals in RM2 dated to 15,504–14,725 cal. BP are the oldest burials so far discovered east of Wallace’s line, with the possible exception of some disarticulated human remains found in Roti to the southwest of Kisar near Timor. The Roti human bones were recovered from levels dated to ~ 23 ka and may be derived from secondary burials^58^. A secondary burial from Liang Lembudu in the Aru Islands dates to 18,000–16,000 cal BP^59^, however, the Aru Islands formed part of mainland Sahul at this time. Further east, in the Pacific, the earliest human remains are dated to 12,300–11,300 cal. BP at the Pamwak site on Manus in the Bismarck Archipelago^60^. Burials closest in age to those at RM2 in Wallacea were found on Alor from Tron Bon Lei in Square B (TBL-1) dated to 11.7–11.3 ka^61–63^, and the Tron Bon Lei Square C burial dated to 17–7 ka^62^. Unfortunately, direct dating of the RM2 human petrous bones failed, and thus the burials had to be dated using shell samples adjacent to Burial 2. Direct dating of human bones in ISEA is often unsuccessful, due to a lack of well-preserved bone collagen in the tropics, and just under half of the burials in ISEA are indirectly dated by associated shell and charcoal^27^.

The RM2 burials, with their clear evidence for the symbolic treatment of the dead, share many similarities with other early burial sites on Java, Borneo and elsewhere in the Wallacean islands, which may suggest that these islands were connected by shared belief systems. Burial orientation varies by site and at RM2, the significance of the orientation of the flexed burials facing south towards Timor-Leste is unknown. On Java, Gua Braholo 6, contemporaneous with RM2, was also flexed and interred on the right side on an east–west axis^57^. Of note is the fact that the Tron Bon Lei individual TLB-1 from nearby Alor Island, dating to 12 ka, was also interred in a primary flexed position, but lying on its left side^61,63^. Large limestone rocks were placed near the head of each RM2 individual (Fig. 7), in similar fashion to the Liang Tebo cave burial on Borneo^53^ and the TLB-1 Tron Bon Lei burial on Alor^63^.

At RM2, the abundance of obsidian and shellfish in the Phase 1 grave fill relative to Phase 2 and 3 indicates the obsidian artefacts were associated with interment, while the shellfish might represent refuse from ceremonial funerary rites^64^. The more specialised focus on a narrower range of shellfish species associated with the Phase 1 grave compared to the more generalist Phases 2 and 3 supports the occurrence of a discrete disposal event at the time of interment. The shellfish remains could therefore indicate ceremonial feasting and the respectful disposal of ritually sensitive fauna in a mortuary context^65^, as part of funerary rites for a male and female potentially bonded together in life. This is the first reported evidence pointing to the use of shellfish in early mortuary practices in ISEA. There are however documented cases of the elaborate and ritualized deposition of shellfish remains in mounds within mortuary contexts in the Torres Strait (to the east of Kisar) during the last 1000 years, which was important for community socialisation, identity and cohesion^66^. The abovementioned grave inclusions at RM2 (shellfish feasting and exotic obsidian), in addition to those at Tron Bon Lei on nearby Alor (fish-hooks, and cobbles coated with red ochre) three millennia later^58,60^ are rare examples of ritualised funerary rites in ISEA during the Terminal Pleistocene. The evidence at RM2 for the ritualised treatment of the dead is suggestive of religious ideology and social hierarchy, however the true nature of the relationship between the mortuary practices at RM2 and belief systems will remain obscured as—beliefs are difficult to identify in the archaeological record in the absence of historical records^66^.

Settlement patterns and artefact analyses at RM2 are consistent with other sites in Wallacea, indicating substantial changes in society and demography in the region post-LGM when sea levels, temperatures, and precipitation were rising dramatically^29–31^. At this time, populations appear to have increased significantly judging by the substantial increase in settlement intensity at a number of sites^1,14,32^. New populations also moved into the region from the east^20^, when obsidian networks^33–36,38^ and burial practices appear in the archaeological record^27^. Eastern Indonesia, post-LGM, saw significant fluctuations between cooler/drier and warmer/wetter conditions as the strength of the Australian-Indonesian Summer Monsoon changed^31^. Unexpectedly, occupation intensity was greater during the cooler drier Bølling–Allerød period 14.6–12.9 ka^29^ at HSE, and declined after 12.8 ka cal. BP, culminating in hiatuses at both HSE and possibly also RM2 during the early Holocene^11,38^. It appears that reef platform stability and its impact on marine resource availability may have influenced the desirability of settlement on Kisar more so than precipitation^11^. This may indicate resettlement of the island, at both HSE and possibly RM2, by the mid-Holocene when higher stable sea levels likely resulted in more stable productive coral reefs^11^.

Maritime mobility and shell adze technology dramatically expanded throughout the Lesser Sunda Islands after 15.5 ka as seen at HSE^38^ and RM2. Exotic obsidian belonging to the source known as 'Group 1’, has been discovered widely dispersed in archaeological sites across the Lesser Sundas, from Timor, Alor, and Kisar between 15,000 and 4000 years ago, indicating persistent mobility over open seas in this region, which may have been related to a trade and exchange network^33–36,38,67^. The source of this obsidian remains unknown, but is likely to derive from one of the nearby volcanic islands in the Banda Arc such as Wetar^38^. Most of the RM2 Group 1 obsidian flakes, were associated with the grave feature of Phase 1, indicating that the people interred at RM2 were linked in this maritime network. This may represent the earliest extensive maritime network in human history^67^ and could have reduced the risks of settlement on Kisar by facilitating relocation to neighboring islands during periods of climate instability or reef bleaching^38^. *Nautilus* shell disc-bead ornaments like those found in RM2 have also been found throughout the Lesser Sunda Islands dating from the Early Holocene, and likely represented symbols of cultural identity and social status across this region^52–54^. This symbolic material culture may have diffused via the same maritime exchange network that moved the Group 1 obsidian^67^. *Tridacna* shell adzes dating to the early to mid-Holocene from RM2, similar to those found from the early Holocene in other sites in Wallacea and elsewhere in ISEA and the Pacific, suggest wider connections with other island regions outside the Lesser Sunda Islands^37^. These have been interpreted as an important technological advance that improved boat construction during a period of increased inter-island voyaging^37^. The disappearance of the Lesser Sunda Islands obsidian exchange network ca. 4000 BP in Wallacea and on Kisar coincides with widespread socioeconomic transformations that were occurring across the region during the dispersal of Neolithic peoples, ceramics, and domesticates between 4000 and 3000 BP^41–43^, indicating it was superseded by a new socio-political strategy.

On Kisar, a greater marine adaptation relative to other sites in the region by the Terminal Pleistocene is supported by faunal data throughout the sequence and stable isotope data from the two RM2 burials. This strong marine reliance was likely critical to human sustainability on such a small island limited in terrestrial resources. Small amounts of finfish remains at RM2 indicates a mixture of angling, netting and spearfishing on the reef platform. However, these were not present in anywhere near the same abundance as at nearby HSE^38^. This could be related to site function with RM2 interpreted as having had socio-ritualised importance versus HSE being a fishing camp closer to shore^38^. Both sites have concentrated shellfish remains documented throughout the sequence. At RM2 these were mostly neritids and chiton, which can be harvested *en-masse*, and were important resources in early sites in Wallacea^1,10^. They have predictable behaviours and are found concentrated on the base of rocks in shallow water and in rock pools in the intertidal zone^68^. Foraging was thus likely conducted along the narrow intertidal reef with minimal planning required^69^, perhaps by women and children as documented ethnographically on islands in the nearby Torres Straight region^70–74^. Dedicated marine foraging is supported by stable isotope δ^13^C values from the individuals interred at RM2. These are similar to those which have been observed for humans at Asitau Kuru ~ 40,000 years ago and the sites of Matja Kuru 1 and 2 ~ 11–4000 years ago and interpreted as heavy marine reliance^23^. This contrasts with isotopic signatures from larger islands that demonstrated diversification towards terrestrial tropical resources by the end of the Pleistocene that likely included a greater consumption of endemic plants^23^. The small lithic assemblage at RM2 may hold clues to the limited exploitation of terrestrial plant resources on Kisar. On Sulawesi, the largest island in Wallacea about 500 km northwest of Kisar, use-wear and residue analyses of ethno-archaeological and archaeological lithic samples indicate flakes similar to those found at RM2 were used to exploit plants^75^. Future use-wear and residue studies of the Kisar lithic assemblage will help to clarify this.

Persistent settlement in some of the most marginal ecological conditions globally, such as the tiny island of Kisar^38^, has been argued to demonstrate the unique adaptive flexibility of our species^76^ when compared to archaic hominins in ISEA^14,15^. What drove this novel ‘plasticity’ remains debated, but the technological change, social networks, and cultural expressions of communication were all likely essential in enabling populations of our species to develop resilience in challenging habitats while maintaining connectivity between populations on a larger scale. Early burial traditions, subsistence strategies, and evidence for social connectivity at RM2 provide further evidence for this, highlighting the diverse contexts of human socio-cultural experimentation during the Late Pleistocene. Further excavations at RM2 are critical to elaborate in greater detail these socio-cultural developments as well as resolve their chronostratigraphic contexts more firmly.

## Methods

### Excavation and radiocarbon dating

A 1 m^2^ pit (SQA) was opened near to the entrance of the cave where the slope flattened. The pit location was chosen for its central location near the opening with optimal light and where human habitation activity was most likely to have been focused. SQ A was hand excavated using trowels in 5 cm intervals (spits) within stratigraphic layers down to bedrock (Spits A1–A9). When a burial was encountered in the east baulk this was extended at the eastern side of SQ A with another 100 × 50 cm pit (SQ B) for the purposes of accessing the complete burial feature for excavation. SQ B was also excavated in 5 cm spits down to the top of the burial (spits B1–B6). When the top of the burial was exposed in both SQ A and B, the excavation was further extended into the southeast baulk using spades to expose its full extent in the burial extension area. Time constraints did not permit any further systematic excavation. Once the burial feature was exposed fully in plan, the burial was excavated separately in two 5 cm spits removing the sediment and exposing the human skeletal remains in situ using brushes, fine wooden skewers and small trowels to reveal the burial position and associated artefacts.

All excavation units, features, and in situ charcoal, shell and artefacts were recorded in 3D using a Leica TS09 total station. Charcoal was also collected from the section walls. These sections were hand drawn on graph paper and digitized later. Charcoal was also recovered in situ during excavation as well as during sieving and sorting. All material from SQ A (spits 1–9), SQ B (spits 1–6) and from the burial feature was first dry sieved through a 1.5 mm mesh and then bagged by spit and feature for transport to the beach where it was further wet sieved through another 1.5 mm mesh. The material from above the burial extension was discarded without sieving due to time constraints. All retrieved material was then dried and sorted into the following categories: bone, shell, lithics, charcoal, seeds, pottery, ochre, shell artefacts.

In situ marine shell and charcoal were dated at the Australian National University Radiocarbon Dating Centre^77^ and Waikato Radiocarbon dating laboratory. All dates were calibrated in OxCal 4.4, using the Marine20^78^ for marine shell, and a mixed U(0,50) curve combining the IntCal20^79^ and SHCal20^80^ curves for charcoal, as recommended for dates from the Inter-Tropical Convergence Zone^80^ (Supplementary Table S1). These radiocarbon dates were put into a multi-phase Bayesian chronostratigraphic model within OxCal 4.4^81^. For charcoal dates, we applied the *Charcoal Plus* t-type Outlier Model with a prior outlier probability of 10%, which is specifically designed to account for the inbuilt age of charcoal, while also allowing for some stratigraphic movement in an archaeological context^82,83^. The *General* t-type Outlier Model with a prior outlier probability of 5% was used for the marine shell date (S-ANU-26609), following commonly used modelling procedures for general archaeological dates^84^. The model was constructed with three sequential Phases within a single sequence corresponding to the three main phases of occupation identified based on a combination of the stratigraphy, cultural assemblages, and radiocarbon dates. Phases were separated by double boundaries to allow for the possibility of significant changes in sedimentation rates and/or gaps (e.g. hiatus) in the chronostratigraphic record.

These radiocarbon dates were put into a chronostratigraphic model within OxCal 4.4^82,83^. The chronostratigraphic model assumes a Poisson (or random) accumulation of sediment^83^, calculated from the available age data by averaging the model over many values of k^84^. The model interpolation rate was set to a single date per spit, and the unit of depth used was in centimeters (cm). For charcoal dates, we applied the *Charcoal Plus* t-type Outlier Model with a prior outlier probability of 10%, which is specifically designed to account for the inbuilt age of charcoal, while also allowing for some stratigraphic movement in an archaeological context^83–85^.

### Mortuary practices

Mortuary practice reconstructions used standard data collection methods in the field^86–88^ and centered on the anatomical composition of human skeletal material and their spatial distribution in relation to grave goods to reconstruct the initial internment conditions (body position, manipulation) through visual aspects of the burials in plan (photographs and drawings). Once the skeletal material was exposed and carefully cleaned of loose sediment, the burials were photographed in situ with datum points plotted in 3D at critical points on the skull, long bones, hands and feet. Both burials were in exceptionally fragile condition, the bones were soft and flaking so excavation and lifting had to be completed with care. Once recorded, elements were individually lifted by hand and placed into bags with bubble wrap and stored in plastic containers for transport for study at the archaeology department laboratory in Universitas Gadjah Mada (UGM), Jogjakarta. Biological characteristics consisting of age, sex, stature, biological affinity, and pathological history are difficult to assess due to the poor condition of these individuals, which are also covered in concreted carbonate. They were assessed using standard bioarchaeological methods^86^ and will be reported in a future publication.

### Faunal analyses

The vertebrate and invertebrate remains were further sorted in the UGM lab by broad taxonomic group for further designation into the lowest taxonomic level possible whether that be order, family, genus or species. Identification and quantification methods followed^1^. In most cases identification could only be made to class, order or family level due to limited reference materials available at UGM. Vertebrates were quantified by Number of Identified Specimens Present (NISP) and weight (g). In the case of invertebrates, previously identified archaeological reference specimens were available at the department of archaeology at UGM for comparison with the RM2 material. Molluscs were quantified by Minimum Number of Individuals (MNI) using a non-recurring element for each taxon.

Diversity and evenness values were calculated for mollusc MNI by taxa using PAST4 software^89^ to determine changes in foraging strategies, whereby diversity or richness (NTAXA) calculates number of taxa exploited and evenness indices including Simpsons, Shannon–Wiener H, Equitability e, and Equitability J, calculate relative abundance of taxa being exploited to detect changes in foraging strategies^49,90^. The higher the evenness value the less diverse the assemblage and the more generalist the foraging strategy, conversely the lower the evenness score the greater the focus of foraging on fewer taxa suggesting a more specialized strategy.

### Human stable carbon (δ^13^C), oxygen (δ^18^O) isotope analysis

Two human teeth, one from each burial, were selected for stable carbon and oxygen isotope analysis. Stable carbon and oxygen isotope analyses of human and faunal tooth enamel has proven an effective method for reconstructing Pleistocene-Holocene human subsistence in Wallacea. In tropical regions, δ^13^C analysis can be used to distinguish reliance on C_3_ resources, which dominate woodland and forests settings and include crops such as rice, versus C_4_ resources, which are commonly tropical grasses including crops such as millet^91–94^. Plants and animals feeding on plants, in closed canopy forest also have lower δ^13^C than other C_3_ plants growing in more open areas thanks to the ‘canopy effect’^95^. Higher δ^13^C in marine producers than all C_3_ terrestrial plants^96,97^ facilitates distinction of marine versus terrestrial C_3_ consumers^98^. While consumption of marine and C_4_ resources can lead to overlapping δ^13^C, the provision of a detailed faunal baseline and contextual information can tease these factors apart^23^. δ^18^O analysis of tooth enamel provides additional mammalian information about imbibed water and consumed food. δ^18^O analysis has also been argued to distinguish terrestrial from marine consumers^99^, though this is not currently evident for Wallacean contexts^23^.

Air-abrasion was used to clean the selected teeth and remove adhering external material. A diamond-tipped drill was used to obtain 8 mg of enamel powder from the full length of the buccal surface of the tooth, representing the entire period of enamel formation. Samples were washed in 1.5% sodium hypochlorite for 60 min, followed by three rinses in purified H_2_O and centrifuging, before 0.1M acetic acid was added for 10 min, followed by another three rinses in purified H_2_O. Samples were subsequently lyophilized for 24 h. 100% phosphoric acid was added to the samples with the gases evolved being measured for δ^13^C and δ^18^O using a Thermo Gas Bench 2 connected to a Thermo Delta V Advantage Mass Spectrometer at the Max Planck Institute for the Science of Human History, Jena, Germany. δ^13^C and δ^18^O values were compared against International Standards (IAEA-603 (δ^13^C = 2.5; δ^18^O = − 2.4); IAEA-CO-8 (δ^13^C = − 5.8; δ^18^O = − 22.7); USGS44 (δ^13^C = − 42.2) and in-house standard (MERCK (δ^13^C = − 41.3; δ^18^O = − 14.4). Replicate analysis of MERCK standards suggests that machine measurement error is *c.* ± 0.1‰ for δ^13^C and ± 0.2‰ for δ^18^O. Overall measurement precision was based on repeat extracts from a bovid tooth enamel standard (n = 20, ± 0.2‰ for δ^13^C and ± 0.3‰).

### *Human and goat tooth enamel strontium (*^87^*Sr*/^86^*Sr) isotope analysis*

Two human teeth, one each from Burial 1 and Burial 2, were analyzed for strontium isotope ratios ^87^*Sr*/^86^*Sr* to investigate human mobility on Kisar. Two goat teeth were also collected from the uppermost layer (Layer 1, Spit 1) for isotopic analyses to compare with the isotopic results from the RM2 individuals. Strontium isotope analysis has been used to assess the mobility and migration patterns of prehistoric humans around the world^100–110^, but little work has focused on applying this method in Island Southeast Asia. The method is based on the premise that the biologically available strontium a human or animal is exposed to during tooth mineralization is retained within the enamel and is resistant to diagenetic alteration in the burial environment. The underlying rocks display variable ^87^Sr/^86^Sr depending on their type, age, and original rubidium (Rb) content. Erosion of the underlying bedrock, airborne dust (loess), sea-spray, atmospheric deposition, groundwater, and stream water are all contributors to the bioavailable strontium. Strontium purified from enamel should be representative of the biogenic strontium available during the time of tooth mineralization^100,104,105^. The ^87^Sr/^86^Sr ratio of tooth enamel reflects the bioavailable strontium isotope signature of the food and drink a person consumed during the time of tooth mineralization^106^.

For radiogenic strontium isotope analysis, two 10–20 mg enamel samples from the same teeth used for the above analyses were chemically prepared within the Class-10 (ISO 4) workstations of the Class 100 (ISO 5) clean laboratory suite of the Centre for Trace Element Analysis, Department of Geology, University of Otago, Dunedin, New Zealand. Prior to transferring to the clean lab, the enamel was cleaned through abrasion with a sonicated Dremel® reinforced diamond cutting wheel to remove possible surface contaminants. Any additional adhering materials, such as organic matter or dentin, were also removed. Samples were then transferred to the clean lab and an appropriate mass of each sample was weighed into an acid-cleaned perfluoroalkoxy alkane (PFA) vial (Savillex Ltd, USA) and digested in 2 ml of 3M HNO_3_ overnight at 110 °C. The digests were then evaporated to dryness then reconstituted in 2 ml of 3M HNO_3_ and subsampled for the determination of strontium concentration by ICP-MS using a 7900 instrument (Agilent Technologies, USA) after appropriate dilution. Strontium was separated from the sample matrix and purified using established protocols in ion exchange chromatography^100,105^. The column eluant containing the strontium was evaporated to dryness, reconstituted in 2% (v/v) HNO_3_, then analysed for its ^87^Sr/^86^Sr composition using a Nu Plasma-HR MC-ICP-MS instrument (Nu Instruments Ltd, UK). All ^87^Sr/^86^Sr data were first corrected for instrumental mass fractionation and isobaric interferences from Kr and Rb across the Sr mass range using standard procedures, then normalized to the composition of the NIST SRM 987 primary standard (^87^Sr/^86^Sr = 0.710248). An additional in-house HPS standard was used to independently monitor the accuracy and external precision of the measurements. All ^87^Sr/^86^Sr results for HPS were in excellent agreement, within error, with the long-term mean value of 0.70762 ± 0.00003 (2 SD, n = 189). Procedural blanks were run with each batch of 48 samples and all yielded negligible Sr levels of < 250 pg.

### Peptide analysis

Enamel peptide preparation followed established methods^111,112^. First the enamel was washed in 3% H_2_O_2_ for 30 s and quenched in MilliQ H_2_O for 30 s. The enamel chip was then conditioned in 60μL of 5% (vol/vol) of a 35% (w/w) HCl solution for 2 min. The HCL conditioning solution was removed and discarded. Another 60 μL of the 5% (vol/vol) HCl was added to the enamel chip and incubated for 2 min for peptide extraction. The HCl extraction solution containing the peptides was then recovered and centrifuged for 5 min at 20,000 rpm to remove any particulates. The extracted peptides were then purified by solid phase extraction on a ZipTip with 0.6 μL C_18_ resin (Merk Millipore, Mass. USA) essentially following the manufacturer’s instructions. In brief, after rinsing of the C_18_ resin in pure acetonitrile (ACN), the ZipTip was equilibrated in 0.1% formic acid (FA) in water before loading the sample of extracted peptides. The bound peptides were then washed on-tip with 0.1% formic acid (FA) in water and eluted in 60% ACN, 0.1% formic acid (FA) in water. The eluate was dried using a centrifugal vacuum concentrator and stored as dried samples at − 80°C.

For peptide analysis by untargeted liquid chromatography-coupled tandem mass spectrometry (LC–MS/MS) samples were reconstituted in 20 μL of 5% ACN, 0.1% FA in water of which 5 μL were loaded into an Ultimate 3000 nano-flow uHPLC system inline coupled to a LTQ-Orbitrap mass spectrometer (Thermo Scientific, Mass. USA). Peptides were separated on a 75 µm ID silica emitter tip (CoAnn Technologies, Wash. Calif. USA) column that was in-house packed with Aeris 2.6 µm PEPTIDE XB-C18 100 Å bead material (Phenomenex, Calif. USA) on a length of 20 cm. The LC gradient between mobile phase A (0.1% formic acid in water) and mobile phase B (0.1% formic acid in 90% aqueous ACN) was developed from 5% B to 25% B over 13 min followed by an increase to 40% B over 3 min. and 90% B over 2 min. at a flow rate of 300 nl/min. The LTQ-Orbitrap mass spectrometer was operated in an untargeted data acquisition mode. The precursor ion scan was performed in a m/z window of 400–2000 at a resolution of 60,000. The precursor ion scan was followed by 9 data-dependent collision-induced dissociation (CID) fragment ion spectrum acquisitions, of which the first three scans were set up as semi-targeted acquisitions of pre-specified precursor masses if present in the precursor ion scan at a threshold intensity of at least 5000 counts. The following 6 CID acquisitions were set up as data-dependent scans of the 6 most intense ions. The peptide targets included in the precursor ion list were three peptidoforms of the AMELY encoded amelogenin isoform (SMIRPPY at m/z 432.23, SM_OX_IRPPY at m/z 440.22 and SM_OX_IRPPYS at m/z 483.74), one peptide specific for the AMELX encoded isoform (SIRPPYPSY at m/z 540.28) and one peptide common to both isoforms (MPLPPHPGHPGYINF at m/z 837.42).

Raw data were analysed by both software-assisted sequence database searches and manual spectrum interpretation. The Proteome Discoverer software (version 2.5, Thermo Scientific, Mass. USA) was used for sequence assignment using the Sequest HT program to search the human reference and UniProt amino acid sequence databases. Enzyme cleavage was set to non-specific and methionine oxidation was included as a variable modification. Precursor and fragment ion intensities (area under the curve) were extracted manually using the Qual Browser program of the Xcalibur software package (version 2.0.7, Thermo Scientific, Mass. USA).

Based on the results of the untargeted data acquisition approach, a fully targeted high resolution multiple reaction monitoring assay was set up for further confirmation of the presence of the AMELY isoform using a 5600 + Triple Time-Of-Flight mass spectrometer coupled to nano-flow liquid chromatography (AB Sciex, Mass. USA). Sample loading and LC conditions were the same as for the untargeted approach. The following precursor ions of selected peptide sequences were targeted. AMELY-specific: SMIRPPY, SM_OX_IRPPY, and SM_OX_IRPPYS; AMELX-specific: SIRPPYPSY, YEVLTPLK; present in both AMELY and AMELX: LPPHPGHPGYINF. Data were analysed with the Skyline software (version 21.2.0, https://skyline.ms/project/home/begin.view?).

### Artefacts

Stone artefacts were identified by raw material with flakes, flake fragments, cores and flaked pieces counted using the total number of stone flaked artifacts [TNA] and the minimum number of flakes [MNF]^113^. Heat damage was present on some stone artefacts and identified by the presence of crazing, potlids and crenulated surfaces. Pottery was counted by NISP and weight and assessed for form and decoration. Shell artefacts were counted, weighed, photographed and recorded by taxa and evidence for use-wear and/or manufacture.

### Obsidian geochemistry

Obsidian artefacts were analysed semi-quantitatively by portable X-Ray Fluorescence analysis (pXRF), a Bruker Tracer III-SD was employed. Manufacturer recommended settings were used of 40 keV and 42 mA using a 0.1524 mm Cu, 0.0254 mm Ti and 0.3048 mm Al filter in the X-Ray path and a 60 s live-time count at 145 FWHM. The raw counts of the pXRF were calibrated using 40 international standards provided by MURR^114^. Each artefact was analysed at two spots. Element concentrations of manganese (Mn), iron (Fe), zinc (Zn), gallium (Ga), thorium (Th), rubidium (Rb), strontium (Sr), yttrium (Y), zirconium (Zr), and niobium (Nb) were calculated. Elemental concentrations were compared to six known obsidian sources in ISEA, four obsidian source regions in the Western Pacific and two identified sources in the Lesser Sunda Islands which are only known by artefact occurrences at sites (Group 1 and 2)^26–29^. Discriminant Analysis (LDA) in the PAST4 software was used for comparison^89^.

Geochemical analysis of 31 obsidian flakes was conducted. Discriminant function analysis (LDA) and bi-plots of the elemental composition of each artefact against the reference samples were used and the statistical analysis unambiguously sourced all samples to the Group 1 source (Fig. 11). The initial LDA of Mn, Rb, Sr, Y, Zr and Nb was successful insofar that 98% of all sources could be excluded as a possible origin. Only the sources of Uliang Bundoc on Luzon, Philippines, and Group 1 obsidian, assumed to be located in the Lesser Sunda Islands, match the geochemistry of the samples. Unfortunately, these two sources plot closely together and an unambiguous sourcing by LDA alone was not possible. However, additional analysis by bi-plots of selected elemental compositions revealed that the Uliang Bundoc source is not a good match for the RM2 samples. Sr and Nb were selected to investigate the differences between Uliang Bundoc and Group 1 (Fig. 11). Uliang Bundoc has significantly lower values in both Sr and Nb, and can be excluded as a possible origin of the artefacts.

### Supplementary Information

Supplementary Information 1.

## Data Availability

All raw data is presented in the supplementary information of this paper.
